# Prevalence and Risk Factors of High Blood Pressure among Adults in Banyuwangi Coastal Communities, Indonesia

**DOI:** 10.4314/ejhs.v30i6.12

**Published:** 2020-11

**Authors:** Erni Astutik, Septa Indra Puspikawati, Desak Made Sintha Kurnia Dewi, Ayik Mirayanti Mandagi, Susy Katikana Sebayang

**Affiliations:** 1 Research Group for Health and Wellbeing of Women and Children, Department of Epidemiology, Faculty of Public Health, Universitas Airlangga, Surabaya, Indonesia; 2 Research Group for Health and Wellbeing of Women and Children, Department of Public Health Nutrition, Faculty of Public Health, Universitas Airlangga, Banyuwangi Campus, Indonesia; 3 Research Group for Health and Wellbeing of Women and Children, Department of Biostatistics and Population Studies, Faculty of Public Health, Universitas Airlangga, Banyuwangi Campus, Indonesia; 4 Research Group of Tobacco Control, Department of Epidemiology, Universitas Airlangga, Faculty of Public Health, Banyuwangi Campus, Indonesia

**Keywords:** Coastal community, Diastolic blood pressure, Systolic blood pressure, Cardiovascular disease

## Abstract

**Background:**

Hypertension is a disease that still a problem in the world. Hypertension is a risk factor for heart disease and stroke mortality. Economic development and an emphasis on coastal tourism may have an impact on public health conditions, such as hypertension. This study aimed to determine risk factors related to hypertension among adults in coastal communities in Indonesia.

**Methods:**

This was a cross-sectional study of 123 respondents between the age of 18–59 years old selected by cluster sampling. This study was conducted among coastal communities in Banyuwangi District, East Java, Indonesia. Data was analyzed using multivariate logistic regression.

**Results:**

Our study showed that the prevalence of systolic and diastolic hypertension among residents of coastal communities were as high as 33.33% and 31.71%, respectively. Increasing age was associated with systolic and diastolic hypertension (OR_systolic_=1.11; 95% CI=1.03–1.19, p=0.01 and OR_diastolic_=1.07; 95% CI=1.01–1.15, p=0.03) after controlling other variables. Respondents with the poorest and richer socio-economic status had higher odds of having systolic and diastolic hypertension compared to respondents with the richest socio-economic status (OR_systolic-poorest_ =12.78; 95% CI=1.61–101.54, p=0.02; OR_systolic-richer_=10.74; 95% CI =1.55–74.37, p=0.02 and OR_diastolic-poorest_=10.36; 95% CI= 1.40–76.74, p=0.02;OR_diastolic-richer_=6.45; 95% CI=1.01–41.43, p=0.05) after controlling other variables.

**Conclusion:**

Being of older age and of the lower in socioeconomic status are significantly associated with increasing risk for systolic and diastolic hypertension in these coastal communities. More studies need to be done in these and other coastal village to help design appropriate health promotion and counseling strategies for coastal community.

## Introduction

Globally, cardiovascular disease accounts for approximately 17 million deaths per year, of which 9.4 million deaths were due to complications of hypertension (1). Hypertension is a risk factor for heart disease and stroke that is responsible for at least 45% of heart disease mortality, and 51% of stroke mortality (1). The Indonesian Basic Health Research in 2018 reported that the prevalence of hypertension in Indonesia was 34.1% of the total adult population, and in East Java Province, it was 13.5% (2). However, Banyuwangi, one of the districts in East Java Province, had a higher prevalence of 33.3% in 2016 (3).

Previous studies showed that the prevalence of hypertension tends to be higher in women, in urban area, in those with low education and in those who are unemployed (4–6). Other studies found that there was a tendency of high incidence of hypertension in coastal communities. The high prevalence of hypertension among coastal communities has previously been suggested to be due to high dietary salt consumed from salted dry fish, a staple diet high in sodium and cholesterol (6–8).

The coastal communities of Banyuwangi Regency are growing rapidly driven by economic development centered on coastal tourism. This rapid pace of development may have a significant impact on the health conditions such as obesity, diabetes and hypertension in the community. As happened in other regions in Indonesia and Asia, the prevalence of obesity, hypertension and diabetes mellitus increases with the increase in the economy. The changing diet due to the need to procure food for tourists may also cause an increase in the prevalence of metabolic diseases such as hypertension in Banyuwangi. Hence, this study aimed to determine the prevalence of hypertension and the factors related to hypertension among adult population living in coastal communities of Banyuwangi District, East Java, Indonesia.

## Methods

**Study design**: This is a cross-sectional study conducted from September to November 2016 in 5 coastal communities in Banyuwangi District (Kampung Mandar, Ketapang, Grajagan, Bangsring, Buluagung), East Java, Indonesia. Ethical permission was obtained from the Ethical Committee of the Faculty of Public Health, Universitas Airlangga in Surabaya.

**Population and sampling**: The study population was communities living in 5 coastal villages, aged between 18–59 years. We used cluster sampling methods with villages as clusters. There were 22 coastal villages of the 52 villages in Banyuwangi District. Then, we selected 5 of the 22 coastal villages randomly for study location. After the cluster was selected, all mothers from the Family Welfare Development Group and their husbands in the village were randomly selected (9). There were 156 respondents invited into the study, and 151 respondents agreed to participate (response rate=96.8%). This sample size was sufficient to detect a 44% difference in the proportion of determinants of increased systolic and diastolic blood pressure with the confidence interval of 95%, power of 90%, design effect of 2 and the possibility of rejection of 25%.

**Data collection**: The data collected were primary data. Structured questionnaires were filled in the village office after obtaining consent from the respondents. Data collection and measurement of waist circumference were conducted by trained data collectors consisting of public health students. The data collectors were trained by the researchers on interview technique, questionnaires administration and measurement technique. They then underwent a field testing in which the acceptability of the questionnaires and data collectors' skills were assessed and errors were corrected. Waist circumference was measured at respondents' navel using Medline non-stretchable measuring tape. Waist circumference was used to determine abdominal obesity. Abdominal obesity was divided into yes (waist circumference ≥90 cm in men and ≥80 cm in women) and no (waist circumference <90 cm in men and <80 cm in women) (10). Blood pressure measurements were taken by an experienced nurse. Systolic and diastolic blood pressure were recorded as the average of two measurements using blood pressure monitor (Omron Hem-7130, Omron Healthcare Co., Japan) while respondents were sitting in constant ambient temperature. Weight was measured using the same digital scale (Seca 869, Seca Asia Pacific), and height was measured by stature meter (Seca 213, Seca Asia Pacific). Systolic blood pressure was divided into ≥140 mmHg (high) and < 140 mmHg (low) (11, 12). Diastolic blood pressure was divided into ≥90 mmHg (high) and < 90 mmHg (low) (11,12). All tools were calibrated prior to testing. The questionnaires were tested for validity and reliability in Kepatihan village, Banyuwangi Sub-district, Banyuwangi, on 1 October 2016, and the test resulted in good validity and reliability with Cronbach alpha of 0.72.

**Data analysis**: We analyzed data from respondents aged 18–59 years old. Covariates tested were age (13,14), sex (14,15), education level (16), occupation (16), abdominal obesity (16), Body Mass Index (BMI) (14,15,17,18), socio-economic status (15,19,20), mental-emotional status (21), family health history (14), smoking status (16), family member smoking status, ethnic group (17,22) and location (23). Age was defined as the last anniversary of the respondent at the time of the study. Sex was divided into male and female. The level of education consisted of lower education (no schooling, no primary school and primary school), middle level education (high school) and higher education (graduated from high school and college). Occupation level was categorized as currently working or not; socioeconomic status was categorized into equally distributed quintiles (poorest, poorer, middle, richer and richest) from wealth index derived from Principal Component Analysis of household ownership of radio, goat, chicken and rice field (24). Smoking habits for both respondents and members of their household were divided into smoking and not smoking. Respondents were considered to have family health history if a member of their family had a history of one of the following diseases: diabetes mellitus, seizures, obesity, heart disease, recurrent headache, stroke and high blood pressure. Mental-emotional condition was measured using Self-Reporting Questionnaire (SRQ) consisting of 20 questions (25). Respondents scoring ≥ 6 were considered to have high score and thus having mental emotional problem. Respondents were considered obese if their waist circumferences were ≥90 cm for males and ≥80 cm for females (26,27). Ethnicity consisted of Javanese, Madura and others (Osing, Bali, Sasak and Bugis).

**Statistical analysis**: Data were analyzed using multivariable logistic regression in STATA 14. Covariate variables that had a relationship with systolic and diastolic blood pressure with p-value of <0.25 were included in the initial multivariable analysis to allow for a possibility that insignificant covariates in the univariable analysis might become significant when adjusted by other variables. Backward method was used to select variables to be retained in the final model. Confounding assessment was done by reentering covariate variables into the model one by one, starting from variables that have the greatest p-value. If the difference in Odds Ratio (OR) of the factors between before and after the covariate was included was greater than 10%, the variable was declared confounding and must remain in the model.

**Ethical clearance**: The study was approved by Ethics Committee, Universitas Airlangga, Indonesia with a decision letter numbered 512-KEPK.

## Results

Of the 151 participants who provided data, we excluded data from 16 respondents who were older than 59 years. Of the 135 remaining observations, 14 were excluded from analysis due to incomplete information (2 observations had missing outcome data and 12 observations had missing covariate information). This resulted in 123(81.46%) observations ready for analysis.

The mean age of respondents was 41.82 ±9.08 years, and the mean BMI was 27.28 ±6.67 kg/m^2^. There were 33.33% of respondents who had systolic hypertension and 31.71% who had diastolic hypertension. The majority of the respondents were females (69.92%), had higher education (54.47%), were employed (65.85%), and married (97.56%). There were 96 respondents (78.05%) with abdominal obesity, and 72.36% had lower mental-emotional status. In addition, 47.97% of the respondents had at least one family member who smoked while 83.74% of respondents did not smoke. The majority of the respondents belonged to Javanese ethnicity (63.41%) (Table 1).

**Table 1:** Characteristics of respondents of the study

	Variables	N	% or Mean±SD
Age			41.82±9.08
BMI (kg/m^2^)			27.28±6.67
Systolic Blood Pressure (mmHg)	≥140	41	33.33
	<140	82	66.67
Diastolic Blood Pressure (mmHg)	≥90	39	31.71
	<90	84	68.29
Sex	Male	37	30.08
	Female	86	69.92
Education	Lower Education	26	21.14
	Middle Education	30	24.39
	Higher Education	67	54.47
Occupation	Not working	42	34.15
	Working	81	65.85
Abdominal obesity (cm)	<90 for men or <80 for women	27	22.95
	≥90 for men or ≥80 for women	96	78.05
Socio-economic status	Poorest	23	18.70
	Poorer	21	17.07
	Middle	28	22.76
	Richer	26	21.14
	Richest	25	20.33
Married status	Married	120	97.56
	Died/divorce	3	2.44
Mental emotional status	Low	89	72.36
	High	34	27.64
Family history	No	71	57.72
	Yes	52	42.28
Family members smoking status	No	64	52.03
	Yes	59	47.97
Smoking status	No	103	83.74
	Yes	20	16.26
Ethnic group	Javanese	78	63.41
	Madura	36	29.27
	Others	9	7.32
Location	Kampung Mandar	19	15.45
	Ketapang	24	19.51
	Bangsring	28	22.76
	Grajagan	30	24.39
	Buluagung	22	17.89

Based on multivariable analysis, factors associated with both systolic and diastolic hypertension were age and socio-economic status after adjustment for abdominal obesity, family history and location variables (Tables 2 and 3). The odds of getting systolic blood hypertension increased 1.11 for every one-year increase in age after controlling for other variables (Table 2: OR= 1.11; 95% CI = 1.03–1.19, p=0.01), while the odds of getting diastolic hypertension increased 1.07 for every one-year increase in age after controlling for other variables (Table 3: OR= 1.07; 95% CI = 1.01–1.15, p=0.03). Respondents belonging to the poorest socio-economic status had 12.78 times greater odds of getting systolic hypertension compared to the richest socio-economic status after controlling for other variables (Table 2:OR_systolic_= 12.78; 95% CI = 1.61–101.54, p=0.02). Respondents whose socio-economic status was the poorest had nearly 10.36 times greater odds for obtaining diastolic hypertension compared to respondents from the richest quintile after controlling for other variables (Table 3: OR_diastolic_= 10.36; 95% CI = 1.40–76.74, p=0.02). In addition, respondents belonging to the richer socio-economic status had 10.74 times greater odds of getting systolic hypertension compared to the richest socio-economic status after controlling for other variables (Table 2: OR_systolic_= 10.74; 95% CI = 1.55–74.37, p=0.02). Respondents whose socioeconomic status was the richer had nearly 6.45 times greater odds for obtaining diastolic hypertension compared to respondents from the richest quintile after controlling for other variables (Table 3:OR_diastolic_= 6.45; 95% CI = 1.01–41.43, p=0.05).

**Table 2:** Factors related to systolic blood pressure

Variables		Univariate (N=123)				Multivariate (N=123)			

		OR^a^	95% CI		P Value	AOR^b^	95% CI		P Value
			Lower	Upper			Lower	Upper	
Age		1.14	1.07	1.21	0.00	1.11	1.03	1.19	0.01
BMI (kg/m^2^)		1.01	0.96	1.07	0.73				
Sex	Male	Ref							
	Female	1.06	0.47	2.41	0.89				
Education level	Lower Education	Ref							
	Middle Education	1.17	0.45	3.03	0.75				
	Higher Education	1.13	0.31	4.07	0.86				
Occupation	Not working	Ref							
	Working	1.39	0.62	3.13	0.42				
Abdominal obesity (cm)	<90 for men or <80 for women	Ref				Ref			
	≥90 for men or ≥80 for women	3.61	1.15	11.26	0.03	2.51	1.61	11.83	0.24
Socioeconomic status	Richest	Ref				Ref			
	Richer	11.45	2.24	59.09	0.00	10.74	1.55	74.37	0.02
	Middle	3.83	0.72	20.55	0.12	3.36	0.46	24.31	0.23
	Poorer	3.59	0.62	20.87	0.15	3.76	0.44	32.21	0.23
	Poorest	17.89	3.37	95.03	0.00	12.78	1.61	101.54	0.02
Mental emotional status	Low	Ref							
	High	0.42	0.16	1.07	0.07				
Family history	Yes	Ref				Ref			
	No	1.95	0.89	4.29	0.10	2.43	0.88	6.75	0.09
Family members smoking status	No	Ref							
	Yes	0.67	0.32	1.44	0.31				
Smoking status	No	Ref							
	Yes	0.62	0.21	1.85	0.39				
Location	Kampung Mandar	Ref				Ref			
	Ketapang	0.35	0.10	1.22	0.10	0.55	0.12	2.54	0.45
	Bangsring	0.05	0.01	0.25	0.01	0.09	0.01	0.69	0.02
	Grajagan	0.29	0.09	0.97	0.09	0.33	0.07	1.59	0.17
	Buluagung	0.33	0.09	1.19	0.09	0.68	0.13	3.59	0.65
Ethnic group	Javanese	Ref							
	Madura	0.46	0.18	1.13	0.09				
	Others	0.80	0.19	3.44	0.76				

a Odds Ratio

b Adjusted Odds Ratio

**Table 3:** Factors related to diastolic blood pressure

Variables		Univariate (N=123)				Multivariate (N=123)			

		aOR	95% CI		P Value	bAOR	95% CI		P Value
			Lower	Upper			Lower	Upper	
Age		1.11	1.05	1.19	0.00	1.07	1.01	1.15	0.03
BMI (kg/m^2^)		0.99	0.93	1.05	0.78				
Sex	Male	Ref							
	Female	0.80	0.35	1.81	0.59				
Education	Lower Education	Ref							
	Middle Education	0.82	0.32	2.11	0.69				
	Higher Education	0.94	0.27	3.36	0.93				
Occupation	Not working	Ref							
	Working	2.16	0.91	5.12	0.08				
Abdominal obesity (cm)	<90 for men or <80 for women	Ref				Ref			
	≥90 for men or ≥80 for women	3.30	1.05	10.32	0.04	1.95	0.49	7.76	0.34
Socioeconomic status	Richest	Ref				Ref			
	Richer	8.43	1.63	43.52	0.01	6.45	1.01	41.43	0.05
	Middle	5.45	1.05	28.32	0.04	5.44	0.82	35.94	0.08
	Poorer	3.59	0.61	20.88	0.15	3.73	0.48	28.81	0.21
	Poorest	12.55	2.38	66.01	0.00	10.36	1.40	76.74	0.02
Mental emotional status	Low	Ref							
	High	0.58	0.23	1.42	0.23				
Family history	Yes	Ref				Ref			
	No	2.05	0.92	4.57	0.08	2.33	0.91	5.98	0.08
Family members smoking status	No	Ref							
	Yes	0.90	0.42	1.92	0.78				
Smoking status	No	Ref							
	Yes	0.68	0.23	2.02	0.48				
Location	Kampung Mandar	Ref				Ref			
	Ketapang	0.67	0.20	2.26	0.52	1.17	0.28	4.94	0.83
	Bangsring	0.09	0.02	0.47	0.00	0.24	0.03	1.62	0.14
	Grajagan	0.64	0.20	2.07	0.46	1.01	0.23	4.47	1.00
	Buluagung	0.63	0.18	2.22	0.48	1.56	0.31	7.73	0.59
Ethnic group	Javanese	Ref							
	Madura	0.41	0.16	1.05	0.06				
	Others	0.84	0.20	3.64	0.82				

a Odds Ratio

b Adjusted Odds Ratio

## Discussion

Our study found that the prevalence of systolic and diastolic hypertension among coastal communities in Banyuwangi District was high at 33.33% and 31.71%, respectively. The final model of multiple logistic regression showed that older age and lower socioeconomic status were the determinants of higher systolic and diastolic blood pressure levels after controlling for other variables. Older people had greater odds of having higher systolic and diastolic hypertension compared to younger ones. Those of the lower socioeconomic status had greater odds of having higher systolic and diastolic hypertension compared to those of the highest socioeconomic status.

Our results on coastal communities reflect similar findings from another study conducted in rural community in Indonesia that showed people of older age had greater risk of hypertension compared to younger ones (13). This study found that people aged 40 years or older had greater risk of developing hypertension compared to those aged 17–39 years, and the risk was most prominent among the age group of 55–59 years (13). Other studies also reported an increase in prevalence of hypertension as age increased (4, 28–30). Due to structural changes that comes with aging, arterial wall loses its flexibility and becomes stiffer. Consequently, systolic and diastolic blood pressure increases due to reduce pulsatility of the arterial wall (31).

A research conducted in low- and middle-income countries found that higher incomes, household assets or social class were positively associated with hypertension in South Asia, but in East Asia and Africa, no associations were detected (20). Our results are also in line with other studies showing that lower socioeconomic status was associated with high blood pressure (15,32). Modifiable socio-economic factors, such as education and employment, were also associated with hypertension. This is in line with the fact that the final stages of the epidemiological transition, the burden of chronic diseases including hypertension shifts from the higher socioeconomic groups to the lower socioeconomic groups (15,33). This may be because awareness of prevention and disease control was better in groups with higher socioeconomic status. In addition, people from the higher socioeconomic status had better access to healthcare.

The strength of this study was that we assessed various determinants, including demographic factors, socioeconomic factors, individual lifestyles, smoking factors in the family, ethnicity and family health history. Studies regarding systolic and diastolic hypertension specifically those focusing on coastal areas are limited. Hence, this study will add to the current limited pool of data on the topic. Together with the results for increased blood glucose level (34), this study can provide information on metabolic syndrome in coastal communities. The coastal communities in our study locations comprised mostly people of Javanese and Maduranese ethnic groups. Our results may be generalized for other coastal communities in Indonesia with similar ethnic profile. However, further studies are needed to cover other coastal communities with other ethnic profiles.

The limitation of this study is its cross-sectional design which does not permit assumption for causality of risk factors with outcome (temporal ambiguity). Another limitation perhaps is the small sample size, although only 81.46% observation ready for analysis is considered good. In addition, waist circumference was measured at respondents' navel which might have underestimated the true waist circumference in this population (35).

The prevalence of hypertension among coastal communities in Banyuwangi was high. Factors related to systolic and diastolic hypertension in these coastal communities were older age and lower socioeconomic status. Our finding implies the need for promotion of healthy lifestyle that would reduce the risk of hypertension, such as healthy diet and improved physical activities among coastal communities. There is currently a national program in Indonesia for the management of chronic disease called Prolanis, which help monitor and ensure continuous treatment for chronic disease at the community health centres. The government of Indonesia can optimize the program by integrating it with hamlet level health posts available widely in Indonesia for maternal and child health services. Hamlet level service will improve health care and health information access of the elderly, especially those from low economic households. In addition, policies to ensure adequate access to healthy food, clean water and energy need to be improved by the government especially for older populations and lower socioeconomic conditions.
